# Atomic force microscopy comparative analysis of the surface roughness of two posterior chamber phakic intraocular lens models: ICL versus IPCL

**DOI:** 10.1186/s12886-021-02039-6

**Published:** 2021-07-14

**Authors:** Juan Gros-Otero, Samira Ketabi, Rafael Cañones-Zafra, Montserrat Garcia-Gonzalez, Cesar Villa-Collar, Santiago Casado, Miguel A. Teus

**Affiliations:** 1 Clínica Rementería, Calle Almagro, 36, Madrid, Spain; 2 Universidad CEU San Pablo, Campus Montepríncipe, Madrid, Spain; 3 Hospital Universitario Príncipe de Asturias, Carretera Meco s/n, Madrid, Spain; 4 Clínica Novovisión, Paseo de la Castellan, Madrid, Spain; 5 Universidad Europea, Madrid, Spain; 6 Facultad de Ciencia e Ingeniería de Alimentos, Universidad Técnica de Ambato, Ambato, Ecuador; 7 IMDEA-Nanociencia, Campus Universitario de Cantoblanco, 28049 Madrid, Spain

**Keywords:** Phakic intraocular lens, ICL, iPCL, Atomic force microscopy, Surface roughness

## Abstract

**Background:**

To compare the anterior surface roughness of two commercially available posterior chamber phakic intraocular lenses (IOLs) using atomic force microscopy (AFM).

**Methods:**

Four phakic IOLs were used for this prospective, experimental study: two Visian ICL EVO+ V5 lenses and two iPCL 2.0 lenses. All of them were brand new, were not previously implanted in humans, were monofocal and had a dioptric power of − 12 diopters (D). The anterior surface roughness was assessed using a JPK NanoWizard II® atomic force microscope in contact mode immersed in liquid. Olympus OMCL-RC800PSA commercial silicon nitride cantilever tips were used. Anterior surface roughness measurements were made in 7 areas of 10 × 10 μm at 512 × 512 point resolution. The roughness was measured using the root-mean-square (RMS) value within the given regions.

**Results:**

The mean of all anterior surface roughness measurements was 6.09 ± 1.33 nm (nm) in the Visian ICL EVO+ V5 and 3.49 ± 0.41 nm in the iPCL 2.0 (*p* = 0.001).

**Conclusion:**

In the current study, we found a statistically significant smoother anterior surface in the iPCL 2.0 phakic intraocular lenses compared with the VISIAN ICL EVO+ V5 lenses when studied with atomic force microscopy.

## Background

The quality of the surface of an intraocular lens (IOL) is relevant because it may affect its optical performance [1]. The roughness of the IOL surface has been associated with the amount of light scattering [2, 3].

The surface roughness is different among different types of IOLs, and those differences are related to the IOL material and polishing during the manufacturing process [1]. In addition to light scattering, differences in the IOL anterior surface roughness have been related to postoperative outcomes such as the rotational stability of pseudophakic toric IOLs [4].

The surface roughness can be measured with various techniques, such as optical microscopy and scanning electronic microscopy [5, 6]; notably, atomic force microscopy (AFM) obtains objective measurements with high resolution on the nanometric scale [1, 7]. Atomic force microscopy topographic surface measurements have been previously used in ophthalmology for corneal [8] and IOL surface roughness studies [1, 9, 10], but there have been no previous studies on the surface roughness of phakic IOLs.

Phakic IOLs are designed to be implanted inside the eyes of young patients for long periods of time, and very good results have been published in long follow-up studies [11], but the surface roughness has not been studied in retro-pupilar phakic IOLs for the most recent commercially available models (ICL V5 and iPCL 2.0). As the surface roughness might interfere with the optical quality and may also be relevant for the amount of friction of the IOL with the surrounding structures, such as the iris, we decided to analyze the anterior surface roughness of commercially available retropupilar phakic IOLs.

## Methods

This is a prospective, experimental study. Four phakic IOLs were used for this study: two Visian ICL EVO+ V5 (Staar) and two iPCL 2.0 (Care Group). All of them were brand new, were not previously implanted in humans and were monofocal with a dioptric power of − 12 diopters (D). It has been previously published that phakic IOL surgery is considered a safer procedure than excimer laser for moderate to severe myopia (− 6 D to -20D) [12]. Given the wide range of IOL power available among that interval, the -12D IOL power was arbitrarily chosen, as it falls among the most frequently implanted phakic IOL power’s in our clinical setting.

### Intraocular lens description

**Fig. 1:**
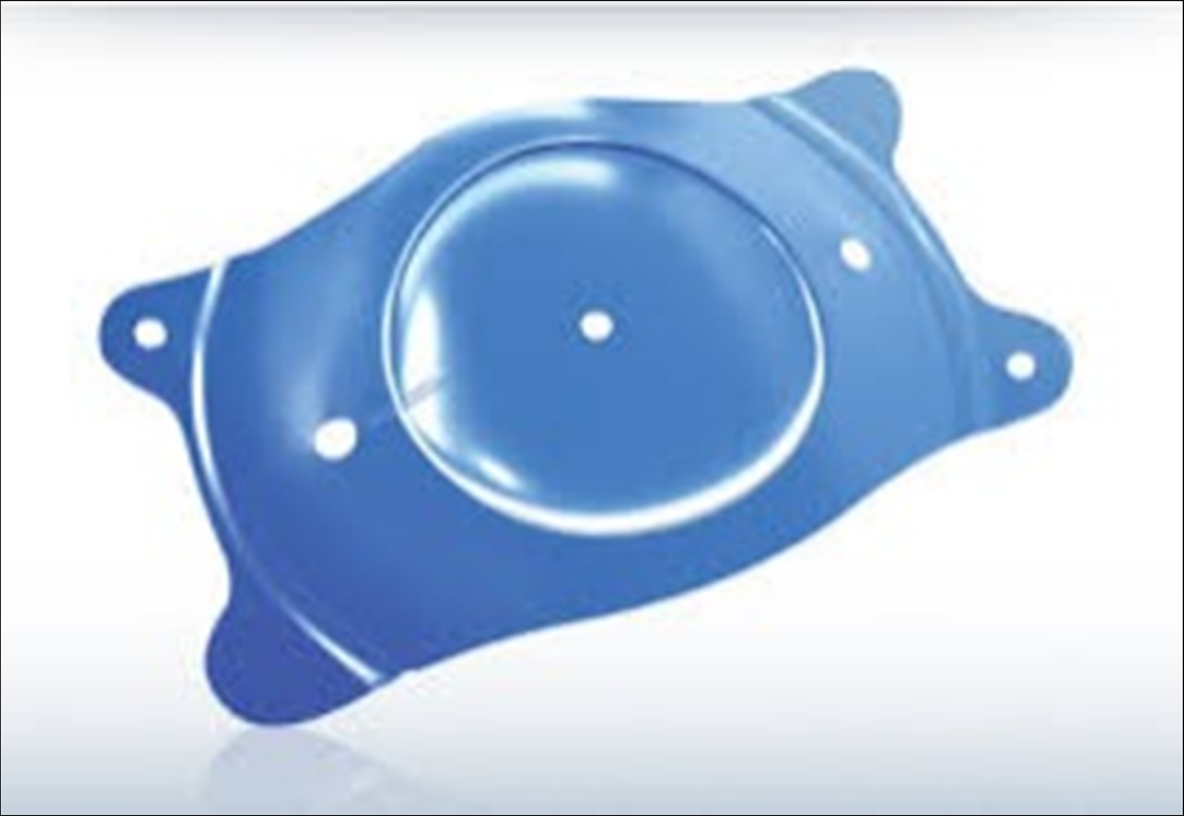
Sample image of Visian ICL EVO+ V5® (Staar) IOL

Visian ICL EVO+ V5® (Staar) (Fig. 1) is a phakic plate-shaped intraocular lens designed to be implanted behind the iris with haptics placed in the ciliary sulcus. It is made of Collamer®, a biocompatible material, and has 4 soft haptics for atraumatic contact with the sulcus. It has 5 holes to ensure an adequate aqueous flow between both sides of the lens: one of them in the optic center, two in the optic periphery, and two in the opposite haptics ends at positions of eleven and four o’clock in the horizontal position to help identify an upside down IOL orientation.

**Fig. 2:**
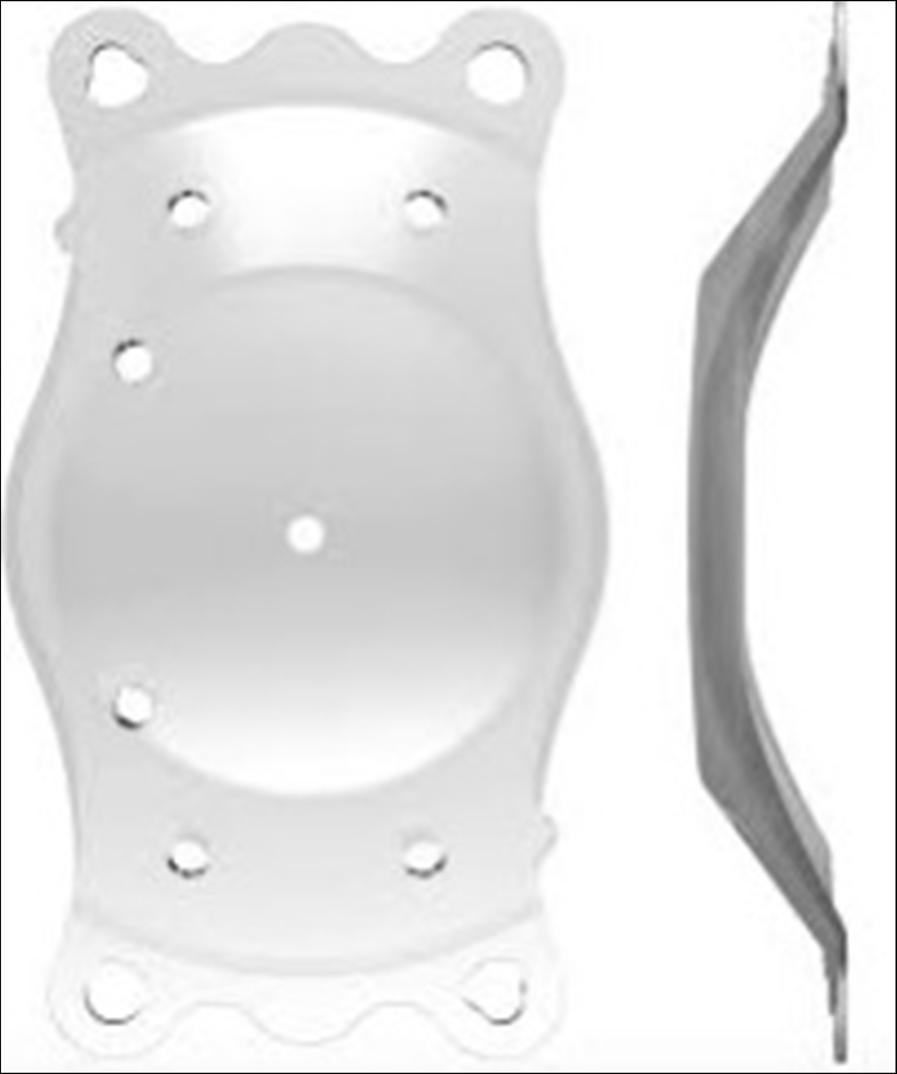
Sample image of iPCL 2.0® (Care Group)

iPCL 2.0® (Care Group) (Fig. 2) is a phakic plate-shaped intraocular lens designed for posterior chamber implantation. It is made of a reinforced hybrid hydrophilic acrylic material with haptics designed to ensure gentle contact with the ciliary sulcus. It has 11 holes designed to maintain an adequate aqueous flow between both sides of the IOL: four of them in the body of the haptic plate, one in the optic center, four in the periphery of the optic and two at the left side of the optic (with the intraocular lens facing up) to indicate proper orientation when implanted.

Additional lens features can be found in Table 1.

**Table 1:** Summary of IOL features

	ICL EVO+	iPCL 2.0
Spherical power range	+10D / -18D	+15D / -30D
Haptics	4	6
Central hole diameter	360 μm	380 μm -420 μm
Lens diameter (mm)	11.6 / 12.1 / 12.6 / 13.2 / 13.7	11 to 14 mm in 0.25 mm steps
Optic diameter	4.9–6.1 mm	6.6 mm
Material	Collamer®	Reinforced hybrid hydrophilic acrylic material
Water percentage	31%	26%

### Atomic force microscopy measurement procedure

Both models of intraocular lenses were originally immersed in buffer media inside the container. Each IOL was removed from the container in ambient air, gently placed on a pristine glass coverslip without touching those areas intended to be analyzed and placed in a milli-Q water bath. Both IOL models are designed to be kept in liquid and we believe they should not be analyzed in dry conditions, i.e. dehydrated, since this could modify their surface features. Also, AFM measurements need to be done in mili-Q water bath and not in the buffer media, since some of the buffer components (salt crystals) could interfere with the silicon cantilever tips displacements needed for measurement.

**Fig. 3:**
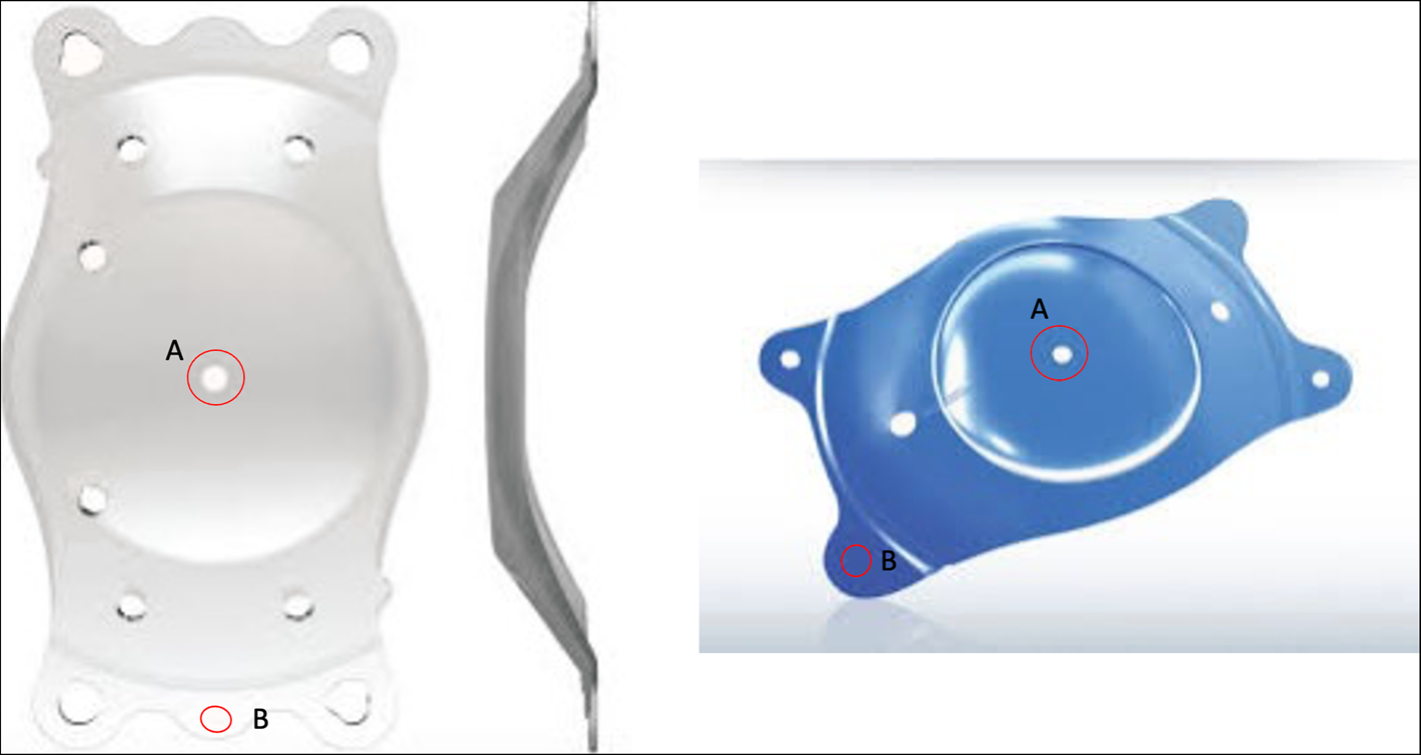
Measured areas map

Atomic force microscopy images were recorded using a JPK NanoWizard II® AFM (JPK Instruments AG (now part of Bruker)) coupled to a Nikon Eclipse Ti-U inverted optical microscope in contact mode and employing Mikromasch HQ:XSC11/Al BS commercial silicon cantilever tips (0.2 N/m, 15 kHz), with a typical 8 nm radius at the end. The same experienced observer, a physicist with no previous knowledge about the design of phakic IOLs performed all measurements. He received the IOL samples without any labelling that might allow him to recognize the IOL manufacturer brand or the dioptric power. The IOL was kept in a milli-Q water bath throughout the AFM analysis. Then, after optical location of the regions of interest on the intraocular lens, the scanning of 10 × 10 μm^2^ areas was performed at 512 × 512 point resolution. Seven areas were evaluated in the anterior surface of each IOL: 4 of them in the central optical zone of the IOL (at locations of twelve, three, six and nine o’clock around the central hole), one at the edge of the central hole and two in the haptics (Fig. 3). The images were processed and analyzed using JPK data processing software. The images obtained have a 512 × 512 resolution for a 10 × 10 μm^2^, which implies around 19.5 × 19.5 nm^2^ for each pixel. Therefore, magnification level can be considered beyond 210·10^6^ nm / (128 · 19.5) nm, that is, beyond 84,134 X*.* We measured the roughness with the root-mean-square (RMS) value within the given areas. The surface root mean square formula was applied over all the images, after having subtracted linearly the background, using the following formula:

$$ {S}_q=\sqrt{\frac{1}{N}\sum \limits_{i=1}^N{z}_i^2} $$

where *z*_*i*_ is the height at each AFM registered point, and *N* is the total number of points (that is, if 512 × 512 resolution, 262,144 points) [13]. We choose the surface root-mean-square roughness analysis because it entails the determination of the whole topographic image roughness at a single pass, avoiding as much as possible any bias. After a minimum modification consisting of a single linear background subtraction at every image, average surface value is prevented for determining any roughness comparison between them. Peak-to-valley roughness may yield misleading results due to spurious peaks during the scanning. Arithmetic roughness analysis could provide valid data. However, root-mean-square roughness analysis enhances the differences between roughness image values, which is more convenient for comparison analysis.

The Mann-Whitney test was used for the comparison of the roughness between both lenses.

## Results

**Fig. 4:**
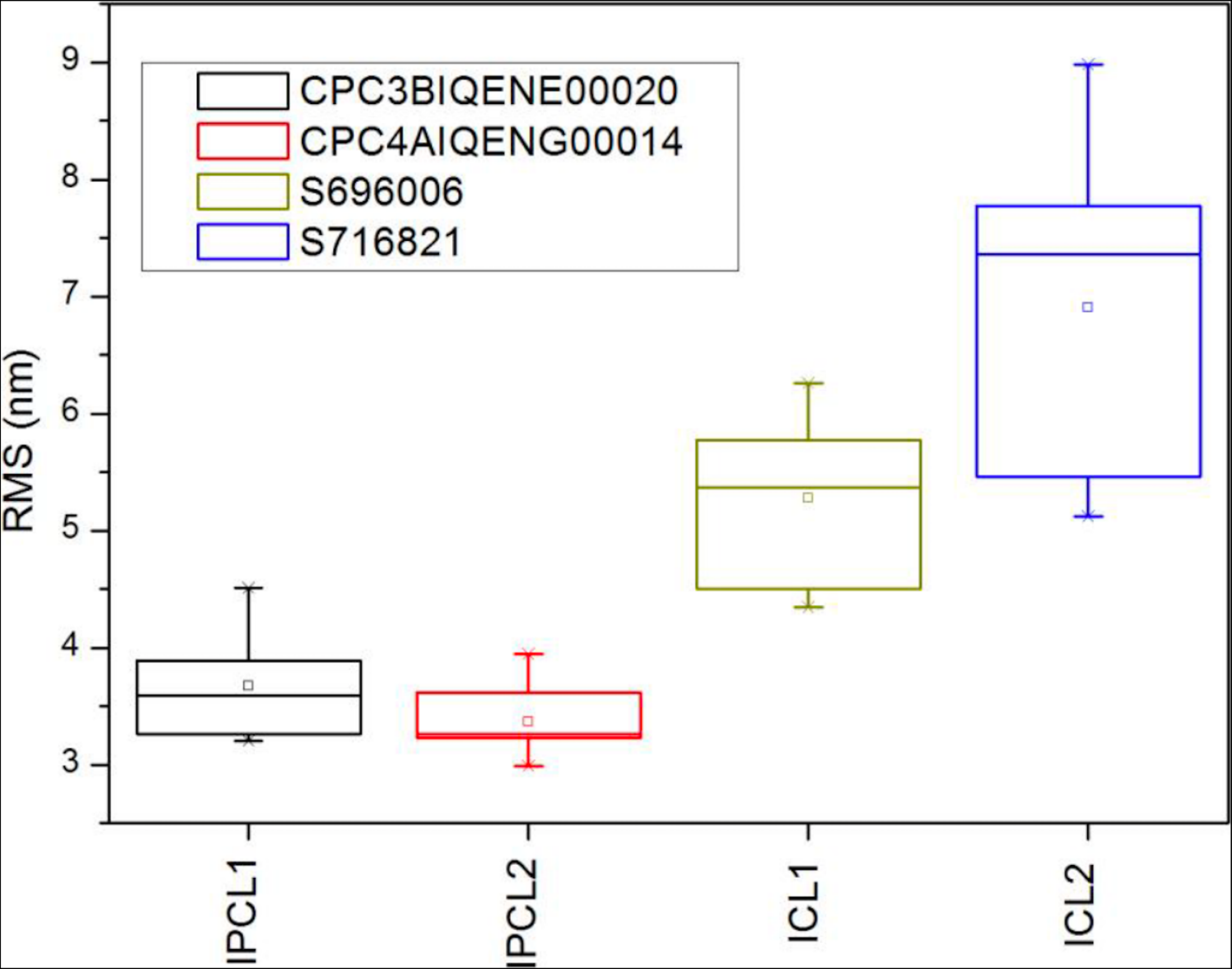
Comparative box and whisker plots of all the RMS values

The mean of all anterior surface roughness measurements was 6.09 ± 1.33 nm in the Visian ICL EVO+ V5 and 3.49 ± 0.41 nm in the iPCL 2.0 (*p* = 0.001). Figure 4 summarizes the comparative box and whisker plots of all the RMS values. In Table 2, we present the mean RMS values of each 10 × 10 μm^2^ area measured for the Visian ICL EVO+ V5 IOLs and in Table 3 for iPCL 2.0.

**Table 2:** RMS values expressed in nanometers (nm) for each 10 × 10 μm^2^ area measured for each Visian ICL EVO+ V5

	IOL 1	IOL 2
Central optical zone 12 h	4.34	6.24
Central optical zone 3 h	4.50	5.12
Central optical zone 6 h	4.71	4.40
Central optical zone 9 h	5.37	5.46
Central hole edge	5.77	7.36
Haptic	6.26	7.43
Haptic	5.44	7.77

**Table 3:** RMS values expressed in nanometers (nm) for each 10 × 10 μm^2^ area measured for each iPCL 2.0

	IOL 1	IOL 2
Central optical zone 12 h	3.89	3.30
Central optical zone 3 h	4.51	3.26
Central optical zone 6 h	3.46	3.25
Central optical zone 9 h	3.37	3.62
Central hole edge	3.82	3.23
Haptic	3.26	3.95
Haptic	3.20	2.99

**Fig. 5:**
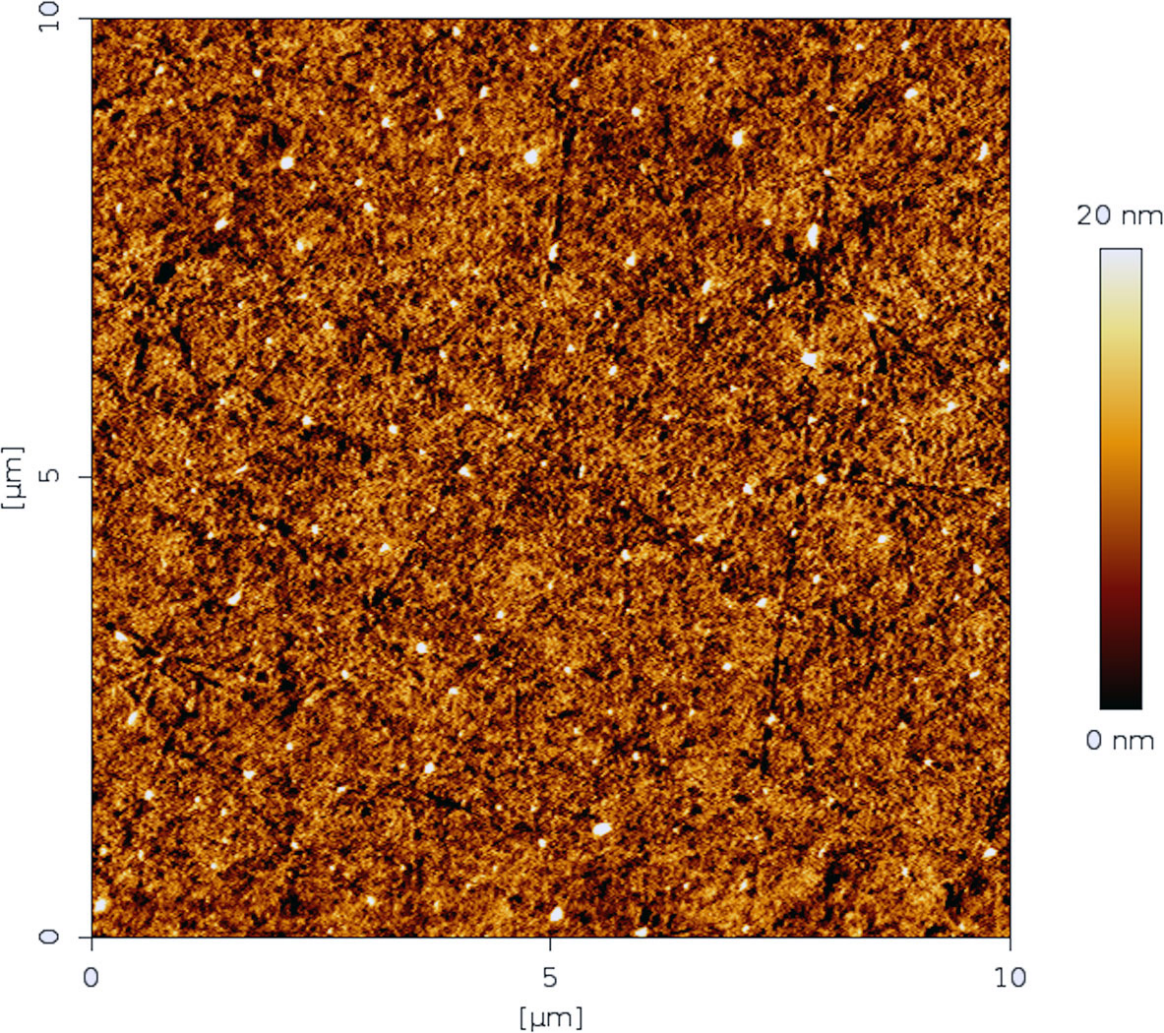
Sample topographic image of a 10 × 10 μm^2^ area measured in an iPCL 2.0 phakic IOL

**Fig. 6:**
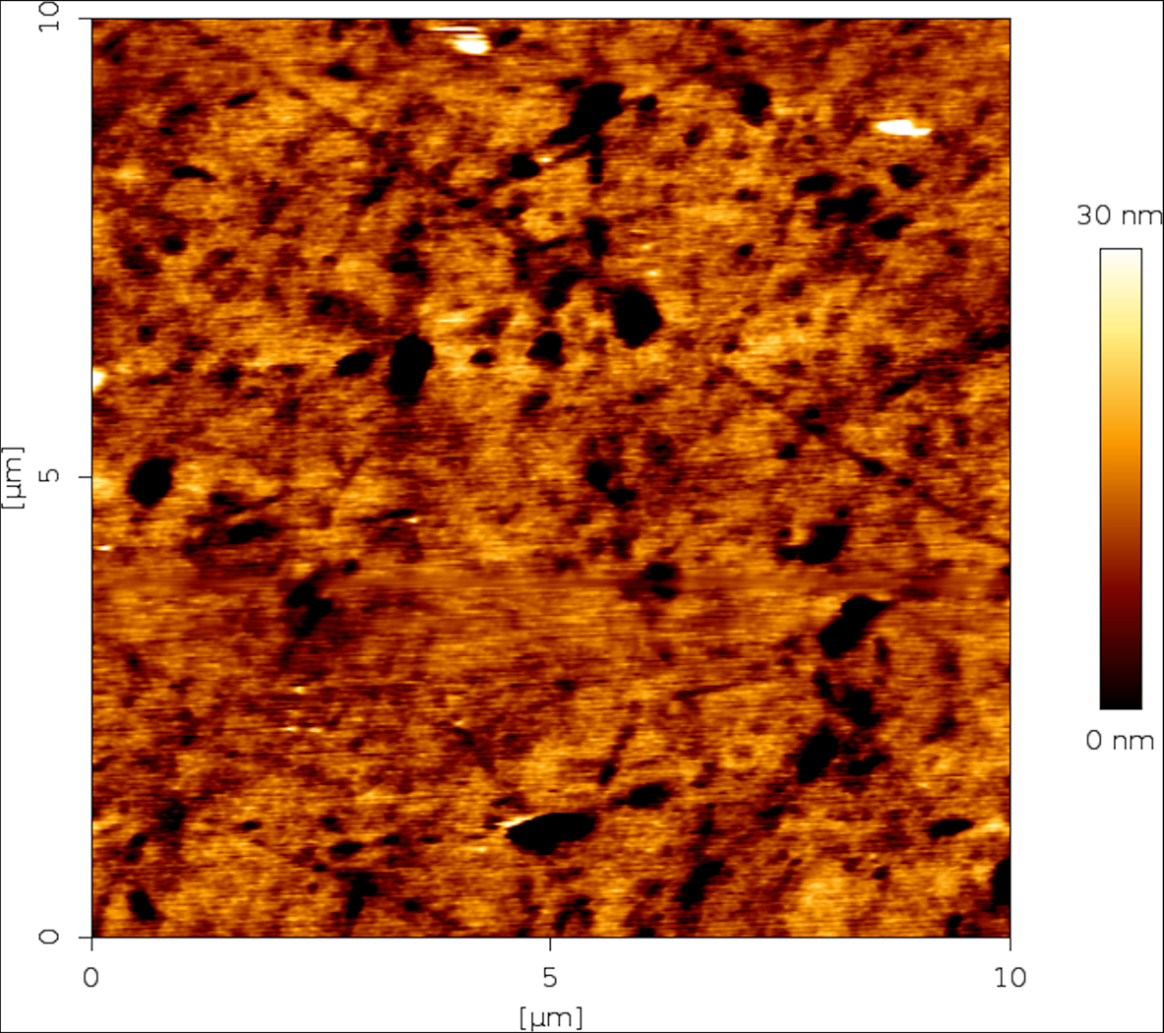
Sample topographic image of a 10 × 10 μm^2^ area measured in a Visian ICL EVO+ V5 phakic IOL

**Fig. 7:**
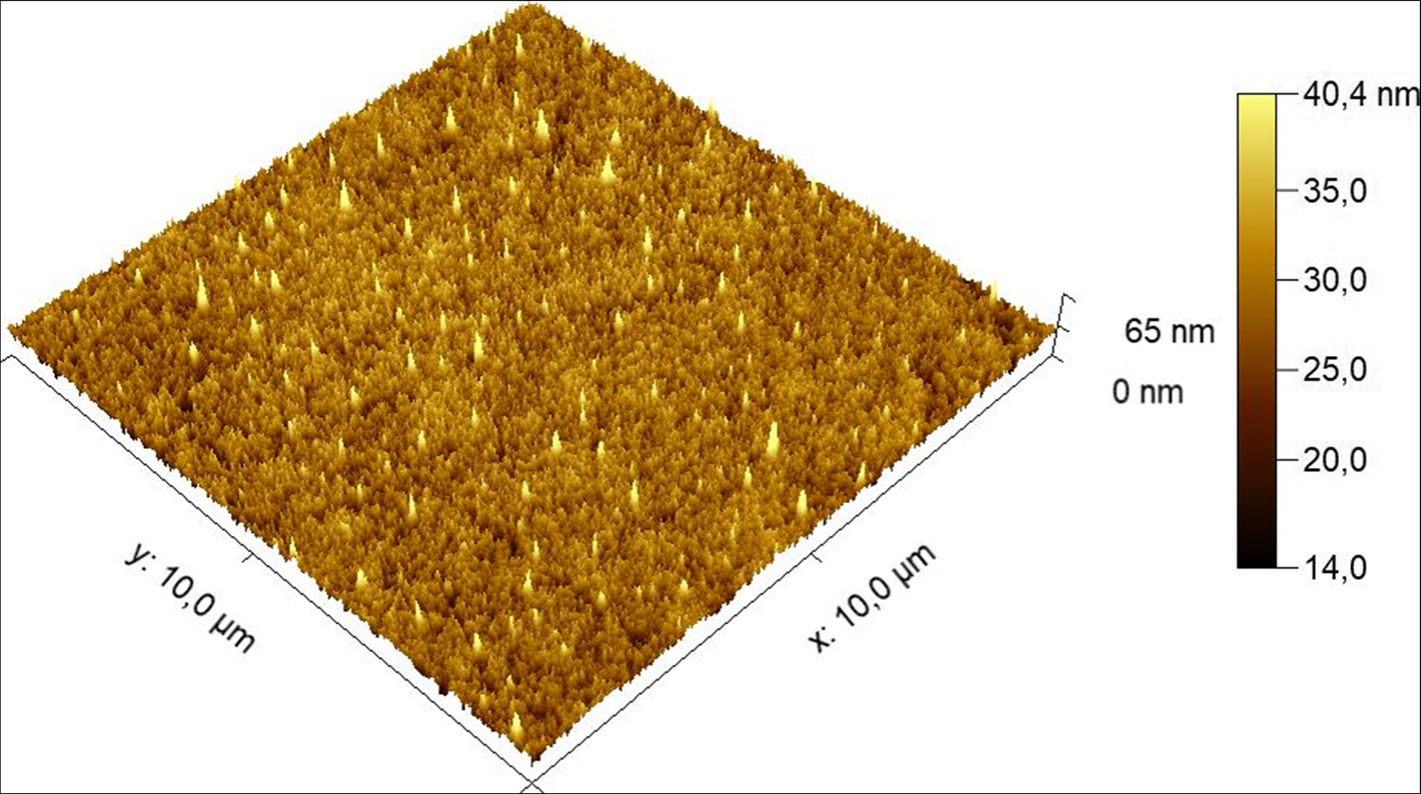
Sample topographic 3-D corresponding to the 10 × 10 μm^2^ surface characterization of an iPCL 2.0 phakic IOL

**Fig. 8:**
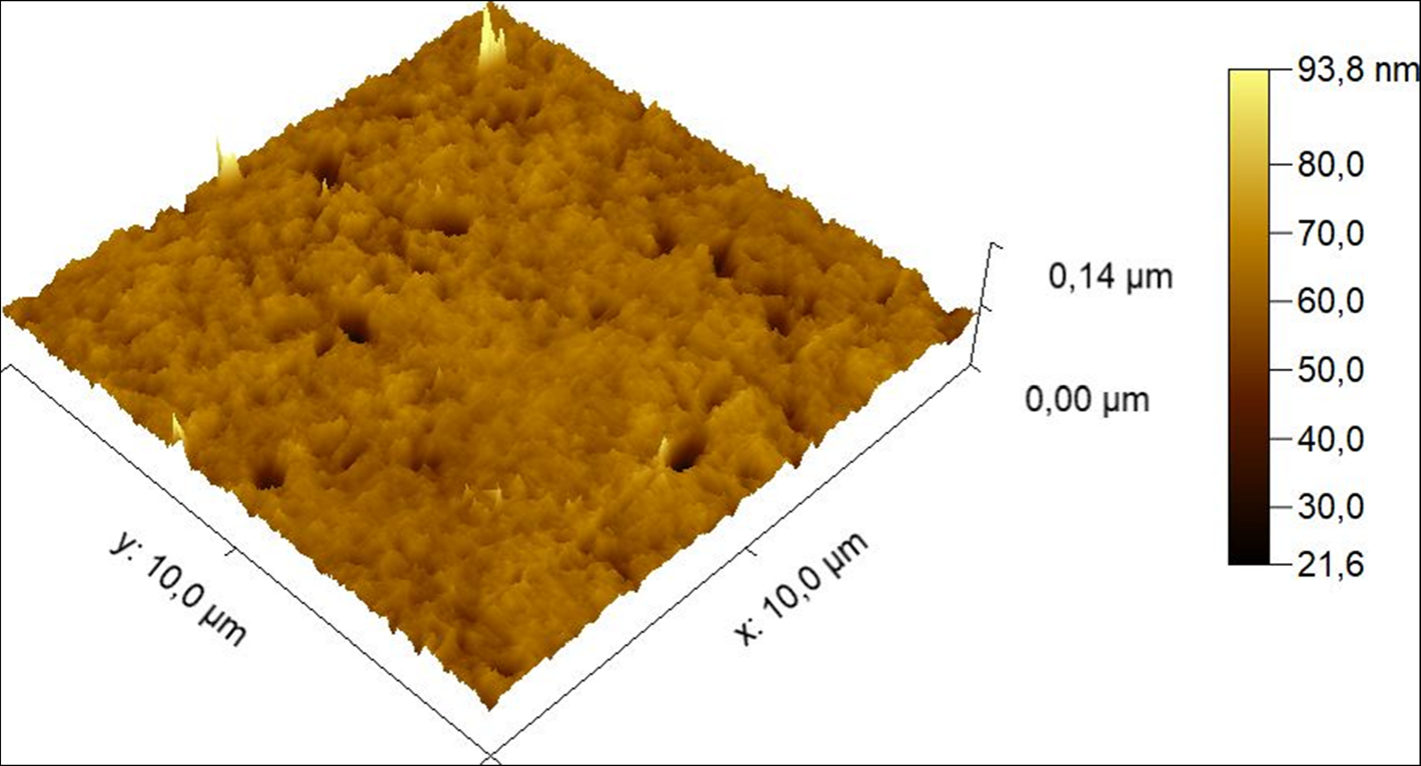
Sample topographic 3-D image corresponding to the 10 × 10 μm^2^ surface characterization of a Visian ICL EVO+ V5 phakic IOL

Figure 5 (iPCL 2.0) and Fig. 6 (Visian ICL EVO+ V5) are sample topographic 2D images of areas measured with both IOL models. Figures 7 and 8 are sample topographic 3D images of areas measured in iPCL 2.0 and Visian ICL EVO+ V5 IOL respectively. In addition to the 3D images, we obtained the following 3D surface parameters:

### iPCL 2.0 phakic IOL image

a- Skewness Ssk [−]: 0.244454; Kurtosis Sku [−]: 2.96521; Maximum peak height Sp [nm]: 37.8 nm; Maximum pit depth Sv [nm]: 27.3 nm; Maximum height Sz [nm]: 65.2 nm; Arithmetic mean height Sa [nm]: 3.0 nm.
b- 3-D parameters: vertical inclination (θ, theta): − 45°; in-plane inclination (φ, phi): 45°; value scale: 0.46, percentage 46% (to fit the same vertical scale as the corresponding image of Visian ICL EVO+ V5 phakic IOL);
c- Scan line discrepancy: 6.9%.
d- Fractal dimension: 2.484 (cube counting).

### Visian ICL EVO+ V5 phakic IOL image

a- Skewness Ssk [−]: − 1.67825; Kurtosis Sku [−]: 14.6036; Maximum peak height Sp [nm]: 75.4 nm; Maximum pit depth Sv [nm]: 60.8 nm; Maximum height Sz [nm]: 136.2 nm; Arithmetic mean height Sa [nm]: 3.5 nm.
b- 3-D parameters: vertical inclination (θ, theta): − 45°; in-plane inclination (φ, phi): 45°; value scale: 1, percentage 100%;
c- Scan line discrepancy: 1.0%.
d- Fractal dimension: 2.21788 (cube counting).

## Discussion

In the current study, we found a statistically significant smoother anterior surface on the iPCL 2.0 phakic intraocular lenses compared with the VISIAN ICL EVO+ V5 lenses when studied with atomic force microscopy.

Although the refractive and safety results achieved with the current models of posterior chamber phakic IOLs are good, mainly with the VISIAN ICL [11, 12, 14, 15], there is a need for a constant improvement in the IOL design and material, as the indications for this kind of implant continue to expand and tend to include younger patients, thus leading to a greater number of patients carrying this implant for a longer time [12]. New data on long-term IOL safety might lead refractive surgeons to adapt their preoperative patient counselling [16].

In recent years, a new model of posterior chamber phakic IOL (iPCL) has been developed and is commercially available in some countries [15, 17]. Regarding the comparison with the current “gold standard”, the Visian ICL, differences in both the material and the design have been previously described, but to the best of our knowledge, there have been no comparative studies searching for differences in the anterior surface roughness between these two IOL models.

From an optical point of view, the IOL surface roughness is a decisive factor determining the amount of light scattering occurring at the surface of the IOL. This phenomenon has been well described for pseudophakic IOLs [1], and it can be hypothesized that it might also occur in phakic IOLs. Whether the differences found in the current study regarding the anterior surface roughness between both IOL groups might induce differences in the amount of light scattering cannot be inferred from our results, but it is plausible to state that the smoother the anterior surface, the less light scattering is induced. The difference in the roughness values at different locations of the same lens found could be related to the polishing process, that seems not be as homogeneous as it should.

Surface roughness differences in pseudophakic IOL’s has been previously studied with AFM [9]. Although surface roughness in phakic and pseudophakic IOL’s might share some facts (i.e. rotational stability or optical disturbances), it can be hypothesized that phakic IOL’s surface roughness has additional potential implications such as the ones derived from the close proximity of the phakic IOL and the iris, the lens or the zonula. The contact between the anterior surface of a posterior chamber phakic IOL and the posterior surface of the iris is dynamic, as it depends on the amount of light entering the eye and the accommodation state [18, 19]. This intermittent iris-IOL rubbing might be one of the reasons behind the well-described tendency for an IOP increase seen with this kind of IOL over time [15]. In fact, both ocular hypertension and pigment dispersion [20] have been described in the long-term follow-up of posterior chamber phakic IOLs, such as the ICL. It should be noted that we cannot directly infer that a higher anterior surface roughness is worse “per se” than a lower anterior surface roughness in terms of ocular tolerance, as the amount of friction between both surfaces (the IOL anterior surface and iris posterior surface) is proportional not only to the surface roughness but also to the elastic modulus of each material involved. The amount of friction is thus determined by several factors, such as the rigidity of the materials, the roughness of both surfaces, and the compression force applied. In addition, these parameters may also vary if measured in dynamic or static conditions. The friction itself has not been analyzed for the phakic IOLs studied here and cannot be directly inferred from our findings. Nevertheless, it is logical to believe that the friction values might be different, as one of the main factors that determines their value (surface roughness) is so different among the IOLs studied. In addition, a higher surface roughness has been previously associated with differences in “friction-related” surgical outcomes in pseudophakic IOLs [4].

Our study has some limitations. First, our small sample size, only two lenses of each IOL model, but since manufacturing processes are expected to be highly reproducible, we believe that our findings do reflect real differences between both IOLs. Second, as previously published [9], surface roughness might be different among different IOL powers, so additional studies are needed in order to confirm if the differences in roughness we found in the current study might be similar in the whole range of IOLs available.

We believe it is of great interest to have more data to better understand the role of the IOL surface roughness and the amount of friction with both the iris and the lens capsule in the long-term safety and optical behavior of posterior chamber phakic IOLs.

## Conclusion

In conclusion, the anterior surface roughness values measured with AFM in the studied phakic IOLs are different, being lower in the iPCL v 2.0 than in the Visian ICL EVO+ V5. The optical and clinical implications of our findings deserve further investigation.

## Data Availability

The datasets used and/or analysed during the current study are available from the corresponding author on reasonable request.

## Abbreviations

IOL: Intraocular lens
AFM: Atomic force microscopy
D: Diopters
RMS: Root mean square
nm: Nanometers
